# Association of Methylenetetrahydrofolate Reductase rs1801133 Gene Polymorphism with Cancer Risk and Septin 9 Methylation in Patients with Colorectal Cancer

**DOI:** 10.1007/s12029-024-01020-y

**Published:** 2024-01-22

**Authors:** Yafei Huang, Ting Su, Qiuting Duan, Xiangcong Wei, Xin Fan, Jinxiu Wan, Luping Liu, Ziqin Dian, Guiqian Zhang, Yi Sun, Tao Zhou, Ya Xu

**Affiliations:** 1 Department of Clinical Laboratory, the First People’s Hospital of Yunnan Province, 157 Jinbi Road, Xishan District, Kunming, Yunnan 650500 P.R. China; 2 The Affiliated Hospital of Kunming University of Science and Technology, The First People’s Hospital of Yunnan Province, Kunming, China; 3 Department of Clinical Laboratory, The Second Affiliated Hospital of Kunming Medical University, 374 Dianmian Avenue, Kunming, Yunnan 650500 P.R. China; 4 Medical School, Kunming University of Science and Technology, Kunming, China

**Keywords:** Septin 9, Colorectal cancer, MTHFR, Methylation, Polymorphism

## Abstract

**Purpose:**

Colorectal cancer (CRC) is one of the most common malignancies, with a high incidence and mortality worldwide. Methylated Septin 9 (mSEPT9) has been used clinically as an auxiliary tool for CRC screening. The aim of the present study was to investigate the association of the methylenetetrahydrofolate reductase (*MTHFR*) rs1801133 polymorphism with the risk of CRC and the methylation status of Septin 9 in CRC.

**Methods:**

Information of 540 patients with a confirmed diagnosis of CRC and with a physical examination were utilized to assess the association of the *MTHFR* rs1801133 polymorphism with CRC and the methylation of SEPT9. *MTHFR* rs1801133 polymorphism was genotyped using polymerase chain reaction (PCR). The commercial Septin 9 Gene Methylation(mSEPT9) Detection Kit was used for plasma SEPT9 methylation analysis.

**Results:**

Among 540 patients, 61.48% were men and the median age was 54.47 ± 13.14. 65.37% of all colorectal tumors developed in the rectum. 195 patients had negative mSEPT9 methylation, while 345 had positive results. 87 individuals with stage I, 90 with stage II, 287 with stage III, and 76 with stage IV colorectal cancer were included in the sample. The results demonstrated that the positivity rate and degree of methylation of mSEPT9 were remarkably higher in patients with more advanced TNM stages than in those with less advanced stages. The frequencies of the *MTHFR* rs1801133 CC genotype and allele C carriers in patients with CRC were significantly higher than those in healthy individuals (*P* = 0.006 and *P* = 0.001, respectively). The positivity rate of the mSEPT9 assay was significantly higher among the *MTHFR* rs1801133 TT genotype and allele T carriers than among the CC and allele C carriers respectively. The *MTHFR* rs1801133 TT genotype and allele T carriers were positively associated with the methylation of SEPT9 (OR = 3.320, 95% CI 1.485–7.424, *P* = 0.003 and OR = 1.783, 95% CI 1.056–3.010, *P* = 0.030, respectively).

**Conclusion:**

In conclusion, individuals harboring the *MTHFR* rs1801133 CC genotype had a higher risk of CRC and the *MTHFR* rs1801133 TT carriers were more susceptible to Septin 9 gene methylation.

## Introduction

Colorectal cancer (CRC) is one of the most common cancers of the gastrointestinal tract. In 2020, there were more than 1.9 million estimated new cases of CRC, with approximately 935,000 deaths due to CRC [1], and its incidence and mortality rates are rising in Asian countries, posing a serious health threat [2].

Single nucleotide polymorphisms (SNPs) are found in a large number of genes linked to different forms of cancer [3]. Risk-associated SNPs for cancer are often found in the genes that control the cell cycle, DNA repair, immunity, and cellular metabolism [4]. SNPs in the promoter region affect DNA methylation, histone modification, transcription factor binding, and promoter activity, such as MMP-1 rs1799750 [5]. SNPs in the intron region frequently influence mRNA splicing, genomic imprinting and chromatin looping, such as IRF4 rs12203592 [6], H19 rs2839698 [7]. SNPs in the 3’UTR region typically impact target gene post-transcriptional levels by changing binding to miRNAs; and exon SNPs on exons may impact protein-protein interactions through changing amino acid sequences, such as *MTHFR* rs1801133 [8]. It may be possible to establish potential biomarkers for determining the long-term risk of CRC by identifying the genetic components that raise an individual’s risk of developing CRC.

The rs1801133 polymorphism of *MTHFR* is the most common mutation that decreases the enzymatic activity of MTHFR in folate metabolism. *MTHFR* rs1801133 polymorphisms can affect its activity, resulting in a reduction in 5-methyltetrahydrofolate production. 5-methyltetrahydrofolate acts as a methyl donor and participates in homocysteine regulation, methylation reactions, nucleotide synthesis, DNA synthesis, and repair [9]. This polymorphism results in hyperhomocysteinemia, reduced folate levels, several cardiovascular diseases, cancer, neural tube defects, pregnancy complications, and psychiatric diseases [10, 11]. In contrast, 5-methyltetrahydrofolate affects carcinogenesis by regulating the methylation of CpG sites in the promoter regions of specific genes [12]. A study on a Chinese population [13] and Iranian women [14] found that *MTHFR* rs1801133 TT and CT genotypes were associated with a higher incidence of breast cancer than the CC genotype. While, *MTHFR* rs1801133 TT has been associated with a lower risk of CRC [15].The *MTHFR* rs1801133 TT genotype has been proven to be associated with global DNA hypomethylation and hypermethylation of tumor suppressor genes [16, 17]. Study on CRC showed decreased LINE-1 methylation in individuals with *MTHFR* rs1801133 CC/ rs1801131 AA [18]. However, the evidence regarding the role of *MTHFR* rs1801133 polymorphism in gene methylation is conflicting. Meanwhile *MTHFR* rs1801133 polymorphism shows marked heterogeneity among different populations and areas. Further studies on the effect of *MTHFR* rs1801133 on the risk of CRC and DNA methylation are needed to be clarified in the local areas.

Chaotic gene-specific DNA methylation is a risk factor for CRC and a promising candidate for biomarker development [19]. The Septin 9 (SEPT9) gene methylation test was approved by the US Food and Drug Administration (FDA) as a screening test for CRC [20]. The SEPT9 gene is located on human chromosome 17q25.3 and contains 17 exons that are involved in cell chromosome separation, DNA migration, apoptosis, and regulation of cell growth. In the early stages of CRC, SEPT9 is methylated and released into the peripheral blood when cancer cells undergo necrosis or apoptosis. By detecting the level of plasma methylated Septin 9 (mSEPT9) in peripheral blood, the risk of CRC in patients can be preliminarily evaluated. A plasma mSEPT9 assay was developed for the early screening and diagnosis of CRC. At present, the sensitivity range of mSEPT9 for CRC diagnosis is 48.2–95.6%, and the specificity range is 79.1–99.1% [20–23]. The sensitivity of the mSEPT9 assay increases with tumor progression from stage I to IV [24]. The mSEPT9 assay also plays a role in monitoring treatment effectiveness and predicting recurrence and survival rates [25]. However, whether related factors affect the methylation of SEPT9 is unclear.

Despite a great deal of research, the processes behind SNPs’ involvement in the genetic susceptibility of cancer are still unclear. Furthermore, variations in epigenetic regulation brought on by gene polymorphisms add to the complexity of SNP-related cancer susceptibility. It is uncertain whether *MTHFR* rs1801133 influences SEPT9 methylation in CRC. The aim of the present study was to investigate the association of the *MTHFR* rs1801133 polymorphism with the risk of CRC and the methylation status of SEPT9 in CRC patients in Yunnan Province in southwestern China.

## Subjects and Methods

### Study Population

To assess the overall prevalence of the *MTHFR* rs1801133 polymorphism, data were obtained from the routine testing of 2,999 patients who underwent *MTHFR* rs1801133 polymorphism testing at the physical examination center of the First People’s Hospital of Yunnan Province from January 2021 to June 2023. The results of *MTHFR* rs1801133 polymorphism and serum homocysteine tests were analyzed.

To evaluate the effect of the *MTHFR* rs1801133 polymorphism on CRC and the methylation of SEPT9, clinical medical information of 540 patients with a definite diagnosis of CRC were collected for the present study from May 2020 to June 2023. And 567 patients from January 2022 to June 2023 who were healthy and just underwent a physical examination for SEPT9 methylation were enrolled. All patients underwent simultaneous examinations of mSEPT9, CEA, CA199, and CA724. Patients with CRC were diagnosed based on pathological results obtained through colonoscopy. Among these 540 patients with CRC and 567 healthy individuals, 140 patients with CRC and 205 healthy individuals were genotyped for *MTHFR* rs1801133. The tumor, node, and metastasis staging (TNM) were defined based on the 8th edition of the Cancer Staging Manual of the American Joint Committee on Cancer [26]. The clinicopathological information of the patients, including age, sex, TNM stage, and tumor location, was collected for analysis. This study was approved by the Institutional Review Board of the First People’s Hospital of Yunnan Province(No. KHLL2023-KY177).

### SEPT9 Methylation Assay

A 10 mL peripheral-elbow venous blood sample was collected using a K_2_EDTA anticoagulant tube. Whole blood was centrifuged (1200 g, 12 min) to separate the plasma within 2 h. After obtaining the plasma, it was centrifuged again for 12 min with the centrifugal force used previously. Then the separated plasma was stored at -20℃, and the test was performed within 4 days. The Septin9 Gene Methylation Detection Kit (Beijing BioChain Co., Ltd.; China) was used to perform the mSEPT9 assay. The experiments were performed in accordance with the manufacturer’s instructions. Real-time polymerase chain reaction (RT-PCR) was performed for 45 cycles to detect both mSEPT9 and the internal control, β-actin (ACTB). A cycle threshold (Ct) ACTB value ≤32, signaled the presence of sufficient DNA and experimental validity. Samples with mSEPT9 Ct values ≤41 were considered positive for mSEPT9.

### *MTHFR* rs1801133 polymorphism Analysis

Genomic DNA from the buffy coat of the K_2_EDTA anticoagulant tube was extracted using a commercial DNA extraction kit (HEALTH Gene Technologies Co., Ltd.; Ningbo, China). Experiments were performed according to the manufacturer’s instructions. Genotyping for *MTHFR* rs1801133 was performed using a mutagenically separated polymerase chain reaction and running on a 3% agarose gel to determine the size, as described by Mokarram [27]. PCR reactions were carried out in a volume of 20 µL using GoTaq Master Mixes (Promega, Madison, USA). The cycling parameters were 5 min at 95℃ followed by 35 cycles of 45 s at 95℃, 1 min at 55℃, and 45 s at 72℃, followed by a single 10-min extension at 72℃. Using a 3% agarose gel,10 µL of each reaction mixture were separated, stained with ethidium bromide, and visualized under UV illumination.

### Statistical Analysis

Data analysis was performed using SPSS software (V.26.0, IBM Inc.; Chicago, Illinois, USA) or GraphPad Prism software (V.9.0; GraphPad Software, Inc.; San Diego, CA, USA). Categorical variables were analyzed using the chi-square test. Numerical data are expressed as mean ± standard deviation and were analyzed using the Student’s t-test. Logistic regression analysis was applied for the risk assessment of CRC and methylation of SEPT9, and the risk of association was estimated using odds ratios (OR) with 95% confidence intervals (95%CI). Statistical significance was set at *P* < 0.05.

## Results

### Frequencies of *MTHFR* rs1801133 Genotypes and Alleles in Yunnan Province

**Fig. 1:**
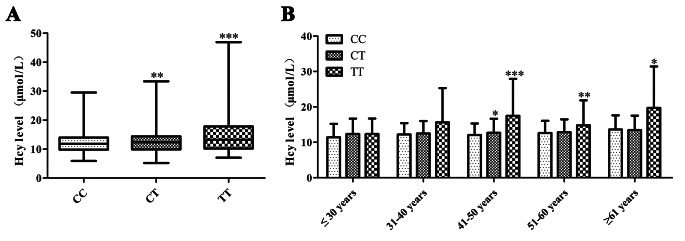
Plasma homocysteine levels. (**A**) Plasma homocysteine levels in *MTHFR* rs1801133 genotypes. (**B**) Plasma homocysteine levels in *MTHFR* rs1801133 genotypes among different age groups. Values are expressed as mean ± SD, *significantly different from CC genotype, *P<0.05, **P<0.01, ***P<0.001

Of the 2,999 patients enrolled, the average age was 46.87 ± 9.78 years. In total, 1,110 (37.0%) patients were homozygous for the wild-type allele (CC) genotype of *MTHFR* rs1801133, 1,435 (47.8%) carried one mutant allele (CT), and 454 (15.1%) were homozygous for the mutant allele (TT). The overall frequencies of the *MTHFR* rs1801133 C and T alleles were 60.9% and 39.1%, respectively. The frequencies of the *MTHFR* rs1801133 polymorphism among the enrolled patients were in Hardy–Weinberg equilibrium. The distribution of the *MTHFR* rs1801133 polymorphism between males and females is shown in Table 1; the frequencies of the T allele and TT genotype among males were significantly different from those of females. The serum homocysteine (Hcy) levels of 2,960 participants were tested. The median Hcy level in patients with the *MTHFR* rs1801133 CC genotype (*n* = 1,096) was 11.80 (9.80–13.97) µmol/L. Patients with *MTHFR* rs1801133 CT (*n* = 1,415) and TT (*n* = 449) genotypes had elevated Hcy levels of 12.3 (9.90–14.40) and 13.2 (10.20–17.80) µmol/L, respectively (Fig. 1A). Considering the relationship between Hcy levels and age, we categorized the enrolled patients into five age groups (Fig. 1B). With increasing age, the Hcy level among people with the *MTHFR* rs1801133 TT genotype was significantly higher, especially in the age group of 41–50 years (Fig. 1B).

**Table 1:** Frequencies of *MTHFR* rs1801133 genotypes and alleles

Gender	No. of subjects	MTHFR rs1801133 genotype, n(%)				Allele frequency		
		CC	CT	TT^a^	95%CI	C	T^a^	95%CI
Males	2162	791(36.6)	1019(47.1)	352(16.3)	14.7–17.8	60.2	39.8	38.4–41.3
Females	837	319(38.1)	416(49.7)	102(12.2)	10.0-14.4	63.0	37.0	34.7–39.4
Total	2999	1110(37.0)	1435(47.8)	454(15.1)	13.9–16.4	60.9	39.1	27.8–40.3

a: The rs1801133 T allele and rs1801133 TT genotype frequencies among males were significantly different from females(χ^2^ = 4.004, *P* = 0.045, and χ^2^ = 7.876, *P* = 0.005, respectively)

### Value of mSEPT9 Assay in the Diagnosis of CRC in Yunnan Province

**Fig. 2:**
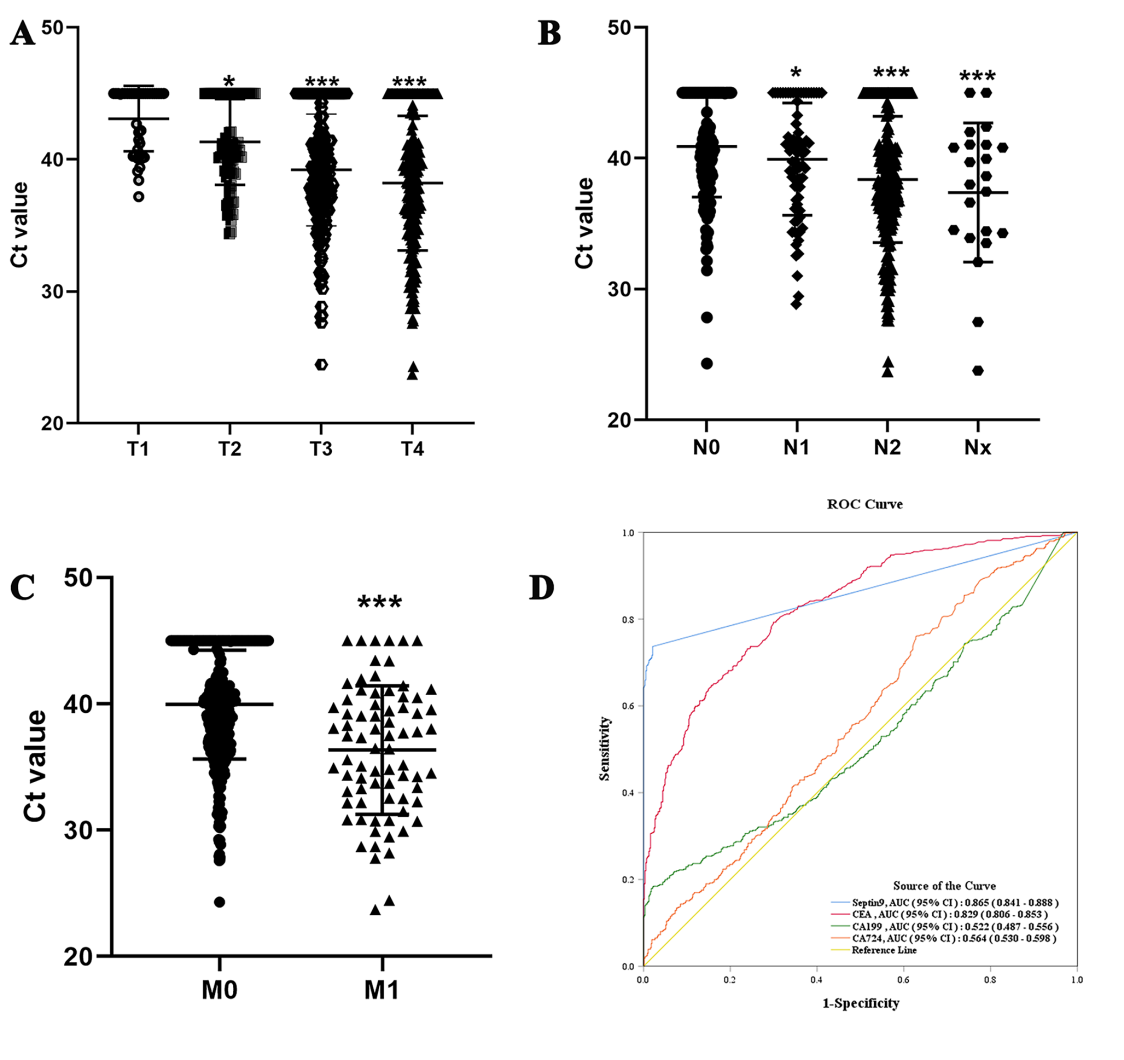
Association Between Septin9 methylation and CRC. (**A**) Ct value of plasma mSEPT9 in each primary tumor (T) category. (**B**) Ct value of plasma mSEPT9 in each primary tumor (N) category. (**C**) Ct value of plasma mSEPT9 in each primary tumor (M) category. (**D**) ROC curves of mSEPT9, CEA, CA19-9, and CA724 in discriminating patients with CRC from healthy participants. Values are expressed as mean ± SD, *significantly different from CC genotype, *P<0.05, ***P<0.001

To assess the diagnostic value of the mSEPT9 assay for CRC, 540 patients with a definite diagnosis of CRC were recruited. Table 2 shows the clinicopathological characteristics of the enrolled patients. Among 540 patients, 61.48% were men and the median age was 54.47 ± 13.14. 65.37% of all colorectal tumors developed in the rectum. 195 patients had negative mSEPT9 methylation, while 345 had positive results. 87 individuals with stage I, 90 with stage II, 287 with stage III, and 76 with stage IV colorectal cancer were included in the sample. The positivity rate of mSEPT9 between male and female patients with CRC was not significantly different (*P* > 0.05). mSEPT9-positive and -negative patients showed no significant differences in age or cancer location (*P* > 0.05). The positivity rate for mSEPT9 was markedly higher in patients with more advanced TNM stages than in those with less advanced stages. The mean Ct values of mSEPT9 in patients with more advanced TNM stages were much lower (Fig. 2A– C), indicating a higher degree of methylation. With respect to other peripheral blood biomarkers, mSEPT9-positive patients had higher levels of CEA, CA199, and CA724 than mSEPT9-negative patients.

**Table 2:** Correlation between mSEPT9 and pathological characteristics of CRC

Variables	Total	S9-positive cases	S9-negative cases	*P* value
**CRC Cases**	540	345(63.9%)	195(36.1%)	
**Gender**				
Male	332	218(65.7%)	114(34.3%)	0.278
Female	208	127(61.1%)	81(38.9%)	
**Age**				
<60years	228	138(60.5%)	90(39.5%)	0.164
≥ 60years	312	207(66.3%)	105(33.7%)	
**Anatomical Location**				
ileocecal region	13	9(69.2%)	4(30.8%)	0.161
ascending colon	45	30(66.7%)	15(33.3%)	
hepatic flexure	24	21(87.5%)	3(12.5%)	
splenic flexure	7	5(71.4%)	2(13.2%)	
descending colon	21	13(61.9%)	8(38.1%)	
sigmoid colon	77	42(54.5%)	35(45.5%)	
rectum	353	225(63.7%)	128(36.3%)	
**Dichotomy Location**				
left sided colon	81	60(74.1%)	21(25.9%)	0.051
right sided colon	104	59(56.7%)	45(43.3%)	
rectum	353	225(63.7%)	128(36.3%)	
**T stage**				
T1	40	12(30.0%)	28(70.0%)	**0.0001**
T2	82	44(53.7%)	38(46.3%)	
T3	228	155(68.0%)	73(32.0%)	
T4	190	134(70.5%)	56(29.5%)	
**N stage**				
N0	182	96(52.7%)	86(47.3%)	**0.0001**
N1	76	42(55.3%)	34(44.7%)	
N2	260	191(73.5%)	69(26.5%)	
NX	13	10(76.9%)	3(36.2%)	
**M stage**				
M0	464	284(61.2%)	180(38.8%)	**0.001**
M1	76	61(80.3%)	15(19.7%)	
**CEA(ng/mL)**				
<5	320	164(51.3%)	156(48.8%)	**0.0001**
≥ 5	218	179(82.1%)	39(17.9%)	
**CA199(U/mL)**				
<43	449	265(59.0%)	184(41.0%)	**0.0001**
≥ 43	87	76(87.4%)	11(12.6%)	
**CA724(U/mL)**				
<6.9	392	236(60.2%)	156(39.8%)	**0.007**
≥ 6.9	144	105(72.9%)	39(27.1%)	

S9: methylated septin9 DNA; CEA: carcinoembryonic antigen; CA199: carbohydrate antigen-19-9; CRC: colorectal cancer

To further understand the performance of the mSEPT9 assay in the diagnosis of CRC, 540 subjects with a definite diagnosis of CRC and 567 subjects who underwent a physical examination for SEPT9 methylation were recruited. The areas under the ROC curve (AUC) for mSEPT9, CEA, CA19-9, and CA724 were 0.865, 0.829, 0.522, and 0.564, respectively (Fig. 2D). The sensitivity and specificity of the mSEPT9 assay (73.89% and 97.88%, respectively) were higher than those of the CEA (79.22% and 70.02%, respectively), CA19-9 (18.40% and 83.90%, respectively), and CA724 (27.00% and 76.68%, respectively) assays.

### Association Between the Risk of CRC and *MTHFR* rs1801133 in Yunnan Province

To investigate the association between the risk of CRC and *MTHFR* rs1801133 in Yunnan Province, among 540 patients with CRC and 546 healthy individuals, 140 patients with CRC and 205 healthy individuals were genotyped for *MTHFR* rs1801133. The frequencies of *MTHFR* rs1801133 CC, CT, and TT in patients with CRC were 50.00%, 38.57%, and 11.43%, respectively. For healthy individuals, the frequencies were 37.80%, 44.86%, and 17.84%, respectively (Table 3), which were similar to the overall prevalence described previously (Table 1). The distribution of *MTHFR* genotypes in these individuals agreed with the Hardy-Weinberg equilibrium. The frequencies of the *MTHFR* CC genotype and allele C carriers in patients with CRC were significantly higher than those in healthy individuals (*P* = 0.006 and *P* = 0.001, respectively). Moreover, Table 3 indicates that CC genotype carriers were associated with a higher risk of CRC before and after adjusting for sex and age.

**Table 3:** Association Between the risk of CRC and *MTHFR* rs1801133 polymorphism

	Control n(%)	CRC n(%)	*P* value	OR(95% CI)	*P* value^a^	OR(95% CI)^a^
Genotype frequency						
CC	69(49.60%)	70(50.40%)		Ref		Ref
CT	95(63.80%)	54(36.20%)	0.016	0.560(0.350–0.898)	0.060	0.550(0.295–1.027)
TT	41(71.90%)	16(28.10%)	0.005	0.385(0.198–0.749)	0.009	0.261 (0.095–0.716)
Allele frequency						
C	233(54.60%)	194(45.40%)		Ref		Ref
T	177(67.30%)	86(32.70%)	0.001	0.584(0.424–0.804)	0.003	0.502(0.320–0.786)

^a^ Adjusted for age and sex

### Association Between SEPT9 Methylation and *MTHFR* rs1801133 in Yunnan Province

To further analyze the association between SEPT9 methylation and the *MTHFR* rs1801133 polymorphism, 135 patients with CRC were analyzed. Among them, 65 patients were negative for the mSEPT9 assay, and 70 patients were positive for the methylation of SEPT9. The positivity rate of the mSEPT9 assay was significantly higher among the *MTHFR* rs1801133 TT genotype and allele T carriers than among the CC and allele C carriers respectively (*P* = 0.040 and *P* = 0.028, respectively) (Table 4). The *MTHFR* rs1801133 TT genotype and allele T carriers had a positive correlation with the methylation of SEPT9 (OR = 3.320, 95% CI 1.485–7.424, *P* = 0.003 and OR = 1.783, 95% CI 1.056–3.010, *P* = 0.030, respectively) (Table 4).

**Table 4:** Association Between Septin9 methylation and *MTHFR* rs1801133 polymorphism

Genotype frequency	S9-negative cases n(%)	S9- positive cases n(%)	Total	*P* value	OR(95% CI)	*P* value^a^	OR(95% CI)^a^
CC	35(53.8%)	30(46.2%)	65		Ref		Ref
CT	27(50.0%)	27(50.0%)	54	0.676	1.167(0.566–2.404)	0.706	1.100(0.699–1.808)
TT	3(18.8%)	13(81.3%)	16	0.018	5.056(1.315–19.439)	0.003	3.320(1.485–7.424)
Allele frequency							
C	97(52.7%)	87(47.3%)	184	0.029	Ref	0.030	Ref
T	33(38.4%)	53(61.6%)	86		1.791(1.062–3.018)		1.783(1.056–3.010)

S9: methylated septin9 DNA; ^a^ Adjusted for age and sex

## Discussion

Both genetic and epigenetic changes are important in CRC pathogenesis. In the present study, the association of the *MTHFR* rs1801133 genotype with CRC risk and SEPT9 methylation in individuals with CRC in the Yunnan Province was reported.

Our study showed that the prevalence of the *MTHFR* rs1801133 CC, CT, and TT genotypes was 37.0%, 47.8%, and 15.1%, respectively. Meanwhile, the mutant T allele carriers had a higher level of serum Hcy, especially among individuals aged 41–50 years. This finding implies that individuals with the mutation in this age range should be cautious of hyperhomocysteine-related disorders. The frequency of the *MTHFR* rs1801133 polymorphism varies among different regions and ethnic groups. The frequency of the mutant T allele is much higher in South America and Asia and less common in Europe and North America. The prevalence of the *MTHFR* rs1801133 TT genotype is 32.2% in Mexico, while in North America, the prevalence ranges from 2.7–10.7% [28]. A study conducted in the United Kingdom showed that the frequencies of the CC, CT, and TT genotypes are 55%, 35%, and 10%, respectively [29]. China is a vast land comprised of multiethnic groups, and the frequency of the *MTHFR* rs1801133 TT genotype varies from 6.4 to 40.8% from south to north [30]. Using larger sample sizes, the frequency of the mutant T allele in Yunnan Province in our study was slightly higher than that reported by Ni et al. [31], and may provide more accurate information on the prevalence of MTHFR polymorphisms in Yunnan Province.

We investigated the association of *MTHFR* rs1801133 polymorphisms with CRC in the local region. Our results showed that the frequencies of the *MTHFR* rs1801133 CC genotype and allele C carriers were significantly higher in patients with CRC and were associated with a higher risk of CRC. Previous studies investigating the association of *MTHFR* rs1801133 and CRC have yielded conflicting results. Three studies conducted in Iran found that the *MTHFR* rs1801133 TT genotypes demonstrated a higher risk of CRC [32–34]. Baghad et al. [35], Slattery et al. [36], Ma et al. [37], and Le Marchand et al [38]. observed similar phenomena. Lin et al. [39] reported that the mutant T allele serves as a predictive factor for CRC in Taiwan. Meta-analyses by Guo et al. [40] and Yang et al. [41] also suggests that the MTHFR mutant T allele is associated with a low risk of CRC in Asians. This disparity may be due to differences in the ethnicity or other factors. To the best of our knowledge, this is the first study to investigate the association of *MTHFR* rs1801133 and CRC susceptibility in the Yunnan Province, China. This finding could help to partially explain why CC carriers have a higher risk of CRC.

In the present study, we demonstrated the diagnostic value of the mSEPT9 assay in the Yunnan Province, with an AUC of 0.865, a sensitivity of 73.89%, and a specificity of 97.88%. The positivity rate and degree of mSEPT9 methylation were remarkably higher in patients with more advanced TNM stages than in those with less advanced stages. These findings are in accordance with those of Sun et al. [42] and Lu et al. [43], who reported that the sensitivity and specificity of the mSEPT9 assay for CRC detection were 73.0% and 94.5%, respectively. Furthermore, we assessed the association of the *MTHFR* rs1801133 polymorphism with the methylation status of SEPT9 in CRC. Our data showed that the *MTHFR* rs1801133 TT genotype and allele T carriers were positively correlated with the methylation of SEPT9. The *MTHFR* mutant T allele has been associated with genomic DNA hypomethylation [44], especially when accompanied by a low folate intake [45]. Supic et al. [46] suggested that the *MTHFR* rs1801133 TT genotype is a risk factor for methylation of the RASSF1A gene in oral squamous cell carcinoma patients. Cheng et al. [47] found a significant association between the *MTHFR* rs1801133 CC genotype and hypomethylation of the IGF-2 gene in transitional cell carcinoma of the bladder. The methylation status of the WIF-1 gene in CRC has also been associated with the *MTHFR* rs1801133 polymorphism [48]. Our study is the first to report that the MTHFR C677T polymorphism contributes to the methylation of the SEPT9 gene in CRC. This finding raises the possibility that *SEPT9* gene methylation influences disease severity and may influence treatment and prognosis in people with colorectal cancer. *MTHFR* rs1801133 gene polymorphism also influences the methylation of *SEPT9*. Our study had some limitations. First, the effects of *MTHFR* polymorphisms are significantly related to the daily folate intake [49]. When analyzing the impact of the *MTHFR* rs1801133 polymorphism on CRC and the methylation of *SEPT9*, we did not consider the folate intake of the enrolled patients. Second, the sample size of patients with CRC remains small, and further studies with larger sample sizes are required to verify our results. Yunnan is a multiethnic province, and research involving multiple centers and ethnic groups can provide more accurate information. Third, because of the limited availability of colonic tissue biopsies, we only evaluated the impact of the *MTHFR* rs1801133 polymorphism on serum cell-free circulating methylated SEPT9. Further research is warranted to investigate the effect of the *MTHFR* polymorphism on the methylation of SEPT9 in tumor tissues.

In conclusion, our results suggest that the positivity rate and degree of methylation of mSEPT9 are significantly associated with the TNM stage in CRC patients. Individuals in Yunnan Province with the *MTHFR* rs1801133 CC genotype have a higher risk of CRC and the *MTHFR* rs1801133 TT carriers are more susceptible to SEPT9 gene methylation. Further well-designed, prospective, multicenter, and multiethnic studies are required to examine the influence of MTHFR polymorphisms in gene methylation and CRC pathogenesis.

## Data Availability

Data can be requested by the corresponding author.
